# A Treatise on Diseases of the Eye

**Published:** 1834-01-01

**Authors:** 


A Treatise on the Diseases of the Eye. By *W. Lawrence*, F. R. S. &c. &c. Octavo, pp. 730. London, 1833.

Mr. Lawrence is so favourably known to the profession that any work of his must receive its consideration and respect. But the present period is ill adapted for the publication of an extensive treatise on the diseases of the eye. The recent appearance of the elaborate production of Mr. Mackenzie has in all probability closed the market against the advantageous introduction of wares of a similar description. But this is not our affair, and we pass to the inspection of the work itself.

It is said by the author to be essentially based on the lectures on the anatomy, physiology, and diseases of the eye, delivered at the London Ophthalmic Infirmary. The subjects are considered in greater detail; the opinions and experience of others are quoted and examined; and cases are introduced for practical illustration, wherever that could be advantageously attempted.

It is not consistent with the plan of this Journal to review or to analyze elementary treatises. However able the author may be, however wide his experience may have been, the mass of what he writes must be familiar to the majority of his well-informed contemporaries. The injurious tongue of political enmity has accused the able author before us, of industry disproportioned to his original observation. His liberal acquaintance with the German language, and, perhaps, with modern continental literature, has furnished a specious pretext for scandal, a pretext that has been amply, if not ungenerously used. It might fairly have been anticipated, that the copious references to foreign authors would have proved an earnest of the candid temper of our own, and that criticism on this point might have laid aside its sting. Turning accidentally to the chapters on amaurosis, we find in about *one* page, a distinct and ingenuous mention of the practice of *nine* authors. Unable to impeach or undermine such candour, the critic has artfully suggested that the mass of opinions and of names betray the laborious compiler, and confer upon the monograph the comprehensive monotony of a dictionary or a cyclopædia. As we do not exactly agree with Voltaire, who, enumerating the sins of the Abbé Trublet, seems to find none so gross as compilation, we dismiss the subject without regret, subjoining only for those who may approve of sarcastic wit, the passage to which we have alluded.

> Au peu d'esprit que le bon homme avait
> L'esprit d'autrui par supplément servait,
> Il compilait, compilait, compilait;
> On le voyait sans cesse écrire, écrire
> Ce qu'il avait jadis entendu dire,
> Et nous lassait sans jamais se lasser.

When we turn again to the valuable and voluminous work before us, we are naturally embarrassed in selecting a portion for the gratification of our readers. As we cannot pretend to have perused its seven hundred closely printed pages, our imperfect acquaintance with its contents may lead us to do inadequate justice to their author.

We will endeavour to collect Mr. Lawrence's experience and opinions on some of the questions connected with diseases of the eye most important in themselves, or most mooted at the present period. There are probably none which have given rise to more keen discussion than the local employment of stimulants and astringents. It may be useful or it may be curious to ascertain the sentiments of Mr. Lawrence on their use.

He observes that when the eye is preternaturally red, when it is weak and irritable, when exertion of it or exposure to light causes watering and pain, although it may be easy while at rest, stimulants and astringents are resorted to with the view of causing the distended vessels to contract, and thus removing what remains of inflammatory excitement. He first adverts to the employment of the vinum opii, recommended and practised by the late Mr. Ware. He observes that were that gentleman's representations correct, we must look on it as a remedy of sovereign virtue. He used it in all cases of ophthalmia, chronic and acute, combined, however, in the latter with the treatment which is ordinarily termed antiphlogistic. Mr. Lawrence, for his own part, would never employ it in acute inflammation, for he thinks it would tend to its increase. And yet his aversion is not unmingled with contempt, for he deems that "it cannot do much mischief." Whilst he thinks that it cannot do much harm in the acute stage, he believes that it will not do much good in the chronic, and between these two stools of insufficiency, he allows the remedy to drop to the ground.

Having alluded, in terms of similar faint praise, to the solutions of alum, of the sulphates of copper and zinc, of the nitrate of silver, of the oxymuriate of mercury, and to other preparations to which we need not more particularly advert, he utters the following equivocal condemnation of the whole.

"It may be observed generally, with respect to all these proposed remedies, that if active treatment be resorted to in the first instance, and followed up steadily, they are not wanted; and if insufficient means have been employed, so that a state of chronic inflammation is produced, this is a complaint which it is extremely difficult to remove, and which is not likely to yield to the vinum opii, or any remedies of that class." 123.

We cannot agree with Mr. Lawrence in the sentiments we have quoted—we cannot allow that active inflammation benefited by antiphlogistic treatment can never be reduced by that treatment, or without it, to a state requiring opposite remedies. How often must the practical surgeon have witnessed such a case as this. A weakly patient has conjunctival inflammation. He is cupped or leeched, and purged, and treated in a debilitating manner. The affection which at first had seemed relieved increases, the vascularity is less vivid but perhaps considerable, and disposition to chemosis is established. Such a patient would be killed before he could be cured by a continuance of the treatment. A drop of the vinum opii, or of some other stimulant or astringent affords immediate relief, and ensures a speedy recovery. Such a case occurred to us a few days back, and the supposition is merely the expression of a fact. We detect in this general aversion to the use as well as the abuse of stimulants the advocate of large bleedings and depletion in erysipelas.

*Catarrhal Inflammation.* Perhaps there are few unimportant complaints more annoying to the patient, or, at times, more productive of ennui to the practitioner, than simple catarrhal inflammation. It is generally treated by astringent or by stimulating applications, and their advocates present very flattering accounts of their success. Consistently with what might be expected, stimulants form no portion of Mr. Lawrence's methodus medendi.

He observes that antiphlogistic treatment is required, but that mild measures will in general be found to be sufficient. What those measures are will be seen from the following extract.

"Venesection is not in general necessary; but in a young subject of full habit with catarrhal inflammation in both eyes, and that severe, a full blood-letting would be proper; in ordinary cases, cupping and leeching will suffice. The bowels should be freely evacuated by an active aperient, or, if the tongue be foul an emetic may advantageously follow the loss of blood. Saline sudorific medicines may then be given, such as the liquor ammoniæ acetatis, with nitre, or tartrite of antimony, and occasional purgatives. The patient should be kept warm, taking plentifully of warm diluent drinks, and no animal food, nor fermented liquor. If blood should have been taken by venesection or cupping in the morning, and the alimentary canal should have been subsequently cleared by an emetic and a purgative, the warm bath, or warm pediluvium may be used at night, and a full dose of Dover's powder (from ten to twenty grains) given at bedtime. The patient will be nearly recovered the next day, or it may be necessary to repeat cupping or leeches, to persevere in low diet, diaphoretics, and purgatives for a few days, and perhaps to apply a blister to the nape. In cases where the inflammatory affection is not considerable, and seems entirely referable to a disordered state of the alimentary canal, it may not be necessary to take blood from the part. An emetic, and an active aperient containing calomel, or the latter alone, may be administered, and followed by mild purgatives, the diet being light.

The best local application in these cases is warm water, or poppy fomentation; these are better than cold lotions in catarrhal inflammation." 156.

Cupping, leeching, blistering, purgatives, emetics, salines, and low living, are prominent items in a list of mild measures. Mr. Lawrence, holding a scarificator in one hand and a basin for a vomit in the other, promises with confidence the same success as those who confide in their nitrate of silver solutions. It is pleasant to observe in the practice of medicine, such uniform good fortune with such various treatment.

### Purulent Ophthalmia of Newly-born Children.

The following remarks upon the cause of this affection appear to be characterized by judgment and experience.

"In a great proportion of cases there is vaginal discharge from the mother, leucorrhœa, and sometimes gonorrhœa. The eyes of the infant are exposed to the contact of these morbid secretions in passing through the vagina; hence has arisen the natural inference that they are affected from the actual contact of this matter, and the tolerably regular appearance of the disease on the third day corroborates this notion of contagious origin from direct application of the morbid matter. I was acquainted with a case, in which a married gentleman contracted gonorrhœa, and communicated the disease to his wife then pregnant. The infant which I did not see till four months after birth, had been affected with purulent ophthalmia; one of its eyes was staphylomatous, and the cornea of the other was considerably nebulous. The affection had been totally neglected, even the attendant accoucheur having stated that it was merely a cold in the eye, which would do well of itself. I have seen some cases of very rapidly destructive purulent ophthalmia in infants, when the mother has had gonorrhœa at the time of parturition. Sloughing of the cornea, with extensive ulceration spreading to the interior, and consequent evacution of the globe, had occurred in these instances before I have seen them. An example of purulent ophthalmia in an infant, where the communication of gonorrhœa to the mother, and the infection of the child's eyes by the vaginal discharge were unequivocal, is related towards the end of this chapter. Although the inflammation was severe, it did not produce the serious effects just alluded to. But, on the other hand, purulent ophthalmia is often seen in the children of healthy mothers, at least, of such as appear perfectly healthy, and deny, when questioned, the existence of vaginal discharge in any shape. As we cannot carry the investigation further, the source of the complaint must remain at least doubtful in such cases, and consequently the contagious origin of the disorder is still open to dispute." 169.

It would seem more common and more certain for a mother affected with gonorrhœa to produce a child who shall have the ophthalmia neonatorum, than for a child affected with this complaint to have sprung from a mother labouring under gonorrhœa. In other words, gonorrhœa, when it exists, seems always, or usually a cause of the ophthalmia, but there are also other causes independent of vaginal discharge. We have seen several females, patients of the Lock Hospital, delivered. Their infants had in every instance the purulent ophthalmia. But we have also seen the ophthalmia neonatorum occur where we could not determine the existence of gonorrhœa, or decided vaginal discharge in the mother.

There is no necessity for particularizing Mr. Lawrence's treatment of this complaint. It differs little from that usually adopted. When the inflammation is considerable he recommends the application of one or two leeches to the red and swollen superior palpebra. Mild aperients—the prevention of agglutination of the lids by means of a little lard—tepid bathing and cleanliness—and the use of astringents on the subsidence of the inflammation, constitute his rational and not peculiar mode of practice.

His heroic treatment of gonorrhœal ophthalmia occupied formerly many pages of this Journal. We will not revert to it now. But strumous inflammation may briefly detain us. Mr. Lawrence remarks, and with obvious justice, that the general treatment of this complaint is a matter of the utmost moment. His directions on the management of the organs of digestion—on purgatives, on tonics, on diet, and on clothing, seem to us to be characterized by great good sense. Though free from exception, they are not possessed of such novelty or originality as to lead us to transcribe them. We may simply remark, that while Mr. Lawrence is an ardent admirer of warm clothing and defence against all vicissitudes of temperature, he has intentionally or unintentionally omitted to mention the application of cold to the surface by sponging, the shower, or the common bath. Yet, much as may be attributed to purgatives, to tonics, or to clothing, there is probably no single medicinal agent so powerful in improving or maintaining the health of young persons or of old, as this which we have mentioned.

The following is his system of local management.

"In the early period of the complaint, especially in cases which approach to common inflammation, and are attended with considerable redness and pain, a white tongue, and hot skin; or at any time when such symptoms may supervene, abstraction of blood by leeches, and their repeated application, may be necessary. In a severe attack, about or soon after puberty, cupping on the temple may be advisable. It may be expedient to administer an active aperient before leeching. Afterwards tartar emetic may be employed, either alone, or in combination with calomel or sulphate of magnesia. This remedy, given so as to produce vomiting or nausea, may sometimes supersede the abstraction of blood. The intolerance of light is not an indication for the use or repetition of leeches. This symptom has sometimes been regarded as a sign of inflammation, and hence depletion has been carried to unnecessary and injurious lengths. It increases the irritability of the organ, and aggravates the local symptoms, which are lessened by tonics and good diet.

Scarification has been recommended; but I have not practised it in these cases. In the commencement of the affection, when the neighbourhood of the organ and the head generally are hot, cold may be applied to the eye with advantage. But more commonly warm water or poppy fomentation is more comfortable to the patient's feelings. When the intolerance of light and spasm of the lids are considerable, they may be relieved by applying a bit of soft flannel, wrung out of a strong decoction of poppies and chamomile-flowers, as warm as it can be borne.

The local employment of opium is resorted to, when the last-mentioned symptoms are severe. The liquor opii sedativus of Mr. Battley is an eligible form. A

dram of it may be added to an ounce of water, to be used tepid; a few drops may be allowed to pass between the lids. The steam of a mixture of tinct. opii, ʒss. with mist. camphoræ, ℥vijss. may be applied to the organ.

Great benefit is derived from local stimuli after the inflammatory symptoms have been removed, and the alimentary canal has been brought into a healthy state. The solution of lunar caustic, from two to six grains to the ounce, dropped between the lids, is the best, and has great influence in diminishing the irritability of the eye, and promoting the cicatrization of ulcers. A stronger solution might be employed, if it could be applied to the ulcerated surface by a camel-hair brush.

The red precipitate ointment to the lids is a useful application.

After evacuations, counter-irritation may be usefully employed, either by blister behind the ear or to the nape; or by the tartar emetic ointment. It is necessary to proceed cautiously with blisters in young subjects; they should not be left on longer than six or eight hours; nor is it safe to irritate the surface with the savine ointment. I have seen fatal mortification ensue from the neglect of these precautions. I prefer the ointment, as a more manageable and effectual means of accomplishing the object.

When change of structure is going on in the cornea, the part becoming red and opaque, mercury must be freely administered; and its beneficial influence in arresting disorganization will be most powerfully manifested, if it should affect the mouth. Calomel, or the hydrarg. c̄ creta may be used, either alone, or in combination with James's powder, or with the pulvis ipecacuanhæ comp."

Mr. Lawrence has not said all that without impropriety he might, on the treatment of strumous ophthalmia. We will not indulge in further comment than this—that we doubt the correctness of his remark on that obstinate and distressing symptom, intolerance of light. He would seem to imply, for he does not very clearly express his opinion, that the symptom in question is lessened by tonics and good diet. We have at present a young child under our care, in whom this has not been the case. The intolerance was severe to an extreme degree, and many plans of treatment were tried without success by others and ourselves. At last we ordered calomel and scammony every night, and senna, with the sulphate of magnesia, every morning. In a few days the intolerance of light had disappeared, or at least had almost subsided.

We may allude, in this rambling and imperfect notice, to Mr. Lawrence's brief, but not inexpressive observations, on the diagnosis and the treatment of glaucoma.

"The discolouration of the pupil arising from glaucoma, and that from cataract, may be distinguished by the tint of colour. In glaucoma it is green or yellowish green, and if we look at the eye laterally, we see no discolouration, whilst in cataract the pupil is grey, or greyish white, and it has the same appearance in whatever direction it is viewed. The loss of vision in glaucoma is not in direct proportion to the change of colour in the pupil; with an inconsiderable change, vision may be entirely destroyed or seriously impaired; but in cataract there is a direct proportion between the degree of opacity and the injury to sight. In cataract, vision is best in a moderate or weak light; but in glaucoma it is most perfect in a strong light, because in glaucoma, as the retina is less sensible, more light is required to make an impression on it.

*Prognosis.*—The prognosis in glaucoma is unfavourable; we have no means of changing that condition of the internal parts on which the loss of transparency depends; we cannot bring back again the natural appearance of the pupil; we cannot restore the vision which has been lost; and all we can expect to do, is to preserve the little sight which remains.

*Treatment.*—BEER says that no treatment will be of any effect in preventing complete amaurosis; but I cannot agree with him on that point. There is congestion in the head, the removal of which is attended with considerable benefit. The treatment must be decidedly antiphlogistic: we must take blood by cupping; give active purgatives, and administer mercury; the patient must be put upon a regulated plan of diet, and avoid using the eye. If this treatment be followed up, we shall prevent the disease from advancing.

In the first place, when there is active congestion with pain, the patient is relieved from his uneasy sensations. The continued prosecution of the plan will not only prevent the disease from advancing, but even improve sight when it is begun at an early period of the affection. After taking blood by cupping, which may be repeated according to circumstances, it is sometimes necessary to persevere for weeks or months in the use of mercury, not carrying it to the extent of salivation, and at the same time carefully regulating the diet. In this way I have seen the swoln and pimpled countenance of a drinker surprisingly altered for the better, with corresponding improvement in the complaint; and in some instances, where glaucomatous discolouration of the pupil has been attended with slow inflammation of the iris, evidenced by adhesion of its margin, and with protrusion of it against the cornea, the disease has been kept in check, and good vision has been preserved for years." 394.

We cannot close the volume, without expressing our high opinion of its general character and merits. It is one which should be placed, and which doubtless will be found, in the library of all who collect and preserve the *best* specimens of modern medical literature. Did we feel inclined to hint a fault, we should say it is too much pervaded by that antiphlogistic energy which characterizes the author of the celebrated paper upon erysipelas.

